# Bone Marrow Oxalosis: Crystal Flowers in the Bone Marrow Garden

**DOI:** 10.4274/tjh.galenos.2021.2020.0557

**Published:** 2021-12

**Authors:** Nabhajit Mallik, Man Updesh Singh Sachdeva

**Affiliations:** 1 Postgraduate Institute of Medical Education and Research, Department of Hematology, Chandigarh, India

**Keywords:** Oxalosis, Crystals, Bone marrow

A 20-year-old male with a known case of chronic kidney disease had been diagnosed with nephrolithiasis at the age of 8 months. Since then, he had recurrent renal stones leading to end-stage renal disease and was scheduled for a renal transplant. A computed tomography scan showed bilateral small kidneys with right-sided nephrolithiasis. Complete blood counts revealed hemoglobin level of 70 g/L, white blood cell count of 8.1x10^9^/L, and platelet count of 395x10^9^/L. Bilateral bone marrow biopsies were performed to investigate the cause of persistent anemia, and one of the cores showed replacement of bone marrow with extensive interstitial and paratrabecular deposition of crystals accompanied by fibrosis (Figures 1a, 100^x^, and 1b-1c, 400^x^). The crystals appeared translucent and rod-shaped, and they were arranged in a rosette-like pattern. They were birefringent under polarized light, consistent with calcium oxalate crystals (Figures 1d-1e, 100^x^, and 1f, 400^x^).

Systemic oxalosis results in the deposition of calcium oxalate crystals mainly in the myocardium, cardiac conduction system, kidneys, bones, or bone marrow [1]. This case demonstrates that although bone marrow examination may not be a routine modality for the diagnosis of hyperoxaluria, it should definitely be considered in young patients with renal failure and childhood recurrent nephrolithiasis who present with cytopenia/refractory anemia.

## Figures and Tables

**Figure 1:**
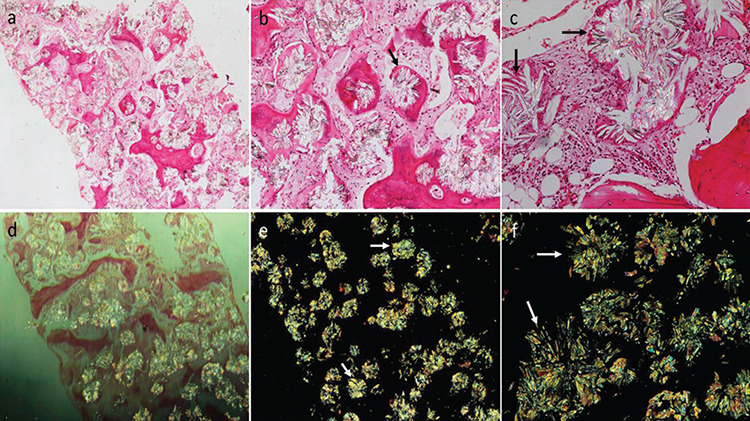
Bilateral bone biopsy core showed replacement of bone marrow with extensive interstitial and paratrabecular deposition of crystals accompanied by fibrosis (a, 100^x^; b and c, 400^x^). Crystals were translucent and rod-shaped, arranged in a rosette-like pattern, and birefringent under polarized light, consistent with calcium oxalate crystals (d and e, 100^x^; f, 400^x^).
